# Inflammation–metabolism composite (CRP–triglyceride–glucose index) predicts all-cause and cardiovascular mortality in MASLD with advanced fibrosis: Evidence from a national NHANES cohort

**DOI:** 10.1097/MD.0000000000049864

**Published:** 2026-07-24

**Authors:** Hao Wang, Yifeng Zhou, Xiaoyu Cui, Yan Wang, Yuan Zhou

**Affiliations:** a Department of Clinical Laboratory, The Affiliated Chuzhou Hospital of Anhui Medical University (The First People’s Hospital of Chuzhou), Chuzhou City, Anhui Province, People’s Republic of China; b Department of Neurology, The Affiliated Chuzhou Hospital of Anhui Medical University (The First People’s Hospital of Chuzhou), Chuzhou City, Anhui Province, People’s Republic of China; c Department of Endoscopy Center, The Affiliated Chuzhou Hospital of Anhui Medical University (The First People’s Hospital of Chuzhou), Chuzhou City, Anhui Province, People’s Republic of China; d Department of General Practice, Wuhu City Second People’s Hospital (Affiliated Wuhu Hospital of East China Normal University); Wuhu City, Anhui Province, People’s Republic of China; e Department of Outpatient Clinic, The Affiliated Chuzhou Hospital of Anhui Medical University (The First People’s Hospital of Chuzhou), Chuzhou City, Anhui Province, People’s Republic of China.

**Keywords:** all-cause mortality, advanced liver fibrosis, cardicovascular mortality, CRP-Triglyceride-Glucose Index (CTI), metabolic dysfunction-associated steatotic liver disease (MASLD)

## Abstract

Metabolic dysfunction-associated steatotic liver disease (MASLD) is highly prevalent and associated with metabolic dysfunction, systemic inflammation, and increased cardiovascular risk. The C-reactive protein–Triglyceride–Glucose Index (CTI) is a novel composite biomarker reflecting low-grade inflammation and insulin resistance. However, its prognostic value for long-term mortality in patients with MASLD and advanced fibrosis remains unclear. Therefore, this study aimed to investigate the association between CTI and the risk of all-cause and cardiovascular mortality in individuals with MASLD using a nationally representative cohort. We used data from National Health and Nutrition Examination Survey 2001 to 2018 and included 8791 adults with MASLD. Advanced fibrosis was defined by FIB-4, NAFLD Fibrosis Score, and AST-to-Platelet Ratio Index. Survey-weighted Cox models evaluated associations between CTI (per standard deviation increase) and all-cause and cardiovascular mortality. Restricted cubic splines examined dose–response patterns. Effect modification by sex, age, and race/ethnicity was assessed in stratified analyses. Over follow-up, 1806 deaths occurred. Higher CTI was independently associated with increased all-cause (HR 1.16, 95% confidence interval [CI]: 1.12–1.20) and cardiovascular mortality (HR 1.12, 95% CI: 1.04–1.20) in MASLD. Among participants with advanced fibrosis, CTI remained associated with all-cause (HR 1.14, 95% CI: 1.07–1.23) and cardiovascular mortality (HR 1.14, 95% CI: 1.01–1.29). Splines showed a U-shaped association between CTI and mortality in MASLD (nadir CTI ~7.5–7.9) but an approximately linear positive association in advanced fibrosis. Associations were stronger in women, older adults, and racial/ethnic minorities. Our observational findings suggest that CTI is associated with long-term all-cause and cardiovascular mortality in MASLD and advanced fibrosis. As a simple, integrative biomarker, CTI may help refine early risk stratification in high-risk MASLD populations.

## 1. Introduction

In early 2020, the term metabolic dysfunction-associated fatty liver disease (MAFLD) was introduced to replace nonalcoholic fatty liver disease (NAFLD), following a 2-stage Delphi consensus process.^[1,2]^ This nomenclature shift emphasizes the central role of systemic metabolic dysfunction in liver disease pathogenesis. MASLD now affects over 30% of the global adult population, and its prevalence continues to rise annually.^[3]^ In addition to accelerating hepatic disease progression, MASLD is associated with severe extrahepatic complications, such as chronic kidney disease, cardiovascular disease, and extrahepatic malignancies – each of which imposes a considerable socioeconomic burden.^[4-7]^

The pathogenesis of MASLD is multifactorial, involving genetic predisposition, metabolic dysregulation, oxidative stress, and dysregulated cytokine signaling.^[8,9]^ M1-type macrophages play a central role in hepatic inflammation and hepatocellular injury by releasing pro-inflammatory cytokines such as TNF-α, IL-6, and IL-1β, which contribute to fibrogenesis.^[10–12]^ Chronic inflammation induces insulin resistance via mediators including TNF and C-reactive protein (CRP).^[13,14]^ Insulin resistance, a key driver of MASLD,^[15]^ promotes hepatic triglyceride synthesis and lipid accumulation,^[16]^ which further aggravates metabolic dysfunction through inflammation, oxidative stress, endoplasmic reticulum stress, and lipotoxicity.^[17]^ These interlinked mechanisms suggest that relying solely on inflammatory or metabolic indices may be inadequate, highlighting the need for integrated biomarkers.

The C-reactive protein–Triglyceride–Glucose Index (CTI), introduced in 2022, integrates CRP and the TyG index to reflect systemic inflammation and insulin resistance simultaneously.^[18]^ CTI has been associated with a variety of health outcomes, including cancer,^[19]^ depressive symptoms,^[18]^ erectile dysfunction,^[20]^ testosterone deficiency,^[21]^ endometriosis,^[22]^ and stroke.^[23]^ However, no previous studies have evaluated the prognostic value of CTI for mortality outcomes in patients with MASLD, particularly in those with advanced liver fibrosis, a population at substantially elevated risk of cardiovascular events and death. Therefore, the present study aimed to investigate the association between CTI and the risks of all-cause and cardiovascular mortality among individuals with MASLD using data from the nationally representative NHANES cohort.

## 2. Materials and methods

**Figure 1.**
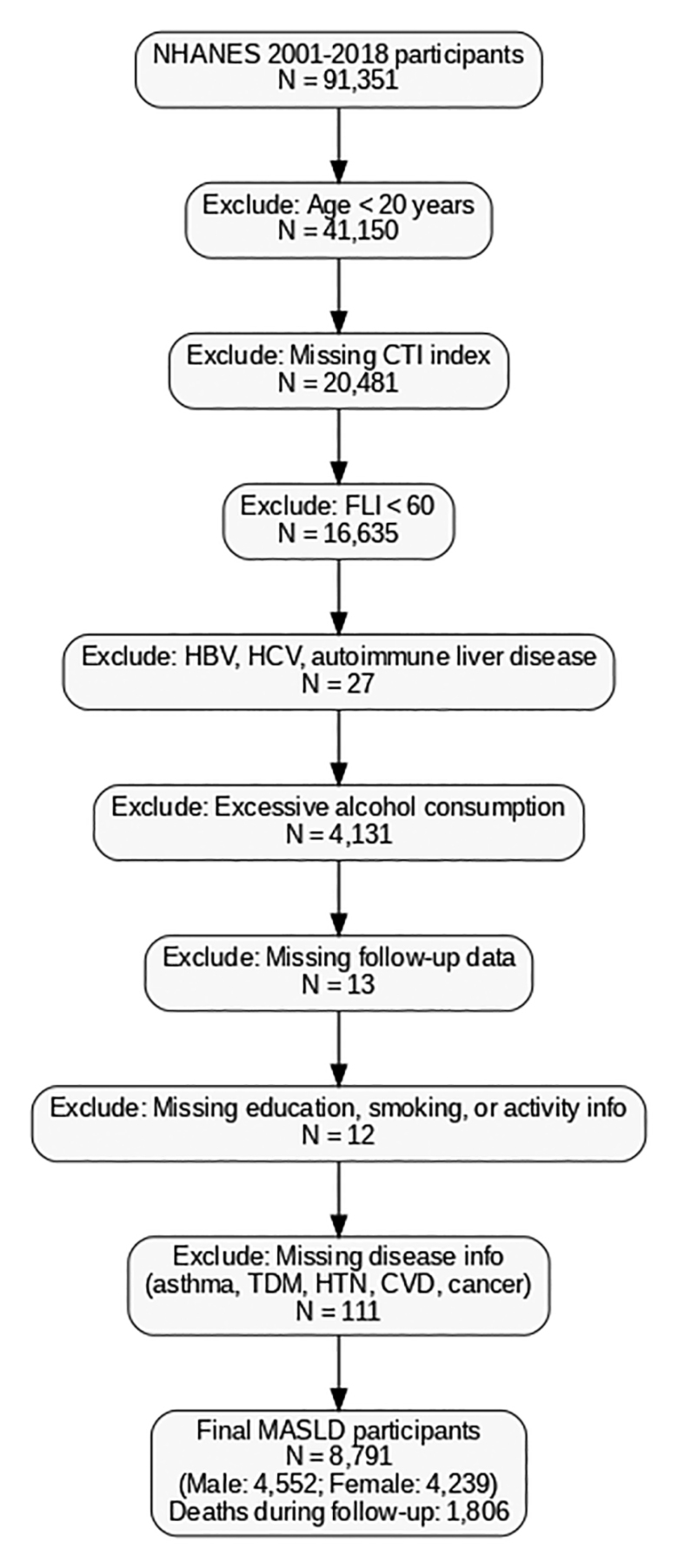
Flow diagram outlining the selection process of study participants. CTI = CRP–Triglyceride–Glucose Index, CVD = cardiovascular disease, FLI = Fatty Liver Index, HBV = hepatitis B virus, HCV = hepatitis C virus, HTN = hypertension, MASLD = metabolic dysfunction-associated steatotic liver disease, NHANES = National Health and Nutrition Examination Survey, TDM = diabetes.

This study is a post hoc retrospective analysis based on data from the National Health and Nutrition Examination Survey (NHANES) conducted between 2001 and 2018. NHANES is administered by the National Center for Health Statistics (NCHS), and all datasets are publicly accessible via its official website (https://www.cdc.gov/nchs/nhanes/). The survey protocol received approval from the NCHS Institutional Review Board, and written informed consent was obtained from all participants. A total of 91,351 individuals were enrolled during the study period. Inclusion and exclusion criteria for the final study cohort are presented in the flowchart in Figure 1.

### 2.1. Calculation of CTI

CTI was calculated using biochemical parameters from fasting blood samples, specifically C-reactive protein (CRP), triglycerides, and fasting glucose levels. The formula was as follows:


$$\begin{array}{c} CTI=0.412\times ln\left[CRP\left(mg/dL\right)\right]\\ +ln\left[\left(triglycerides\times fasting\;glucose\right)/2\left(mg^{2}/dL^{2}\right)\right] \end{array}$$

### 2.2. Definition of MASLD and liver fibrosis

Due to the lack of imaging-based assessment of hepatic steatosis in most NHANES cycles, the Fatty Liver Index (FLI) was adopted as a surrogate marker for hepatic steatosis. Although vibration-controlled transient elastography (FibroScan) data are available in NHANES, these measurements were only collected in the 2017–2018 cycle and therefore were not available for the majority of participants included in the present analysis (2001–2018). Using FibroScan data would have substantially reduced the sample size and statistical power of the study. Therefore, FLI – an extensively validated and widely used noninvasive index for detecting hepatic steatosis in epidemiological studies – was applied to define hepatic steatosis in this large population-based cohort. FLI is a validated and widely used tool for estimating hepatic steatosis, calculated according to published algorithms.^[24,25]^ An FLI ≥ 60 indicates a high probability of hepatic steatosis. According to the Delphi Consensus, MASLD is diagnosed by the presence of hepatic steatosis plus at least 1 cardiometabolic risk factor.^[26,27]^ Other causes of liver disease – including viral hepatitis, autoimmune or genetic liver disorders, drug-induced liver injury, and alcohol-related liver disease (defined as ≥30 g/d in men or ≥20 g/d in women) – must be excluded.^[25]^ The severity of liver fibrosis was assessed using noninvasive serologic scoring systems, including the NAFLD Fibrosis Score (NFS), Fibrosis-4 Index (FIB-4), and the AST-to-Platelet Ratio Index, calculated using validated algorithms.^[25]^ Advanced fibrosis was defined by meeting any one of the following thresholds: AST-to-Platelet Ratio Index > 1, FIB-4 > 2.67, or NFS > 0.676.^[26,28,29]^

### 2.3. Outcome measures

The primary outcome of this study was all-cause mortality in individuals diagnosed with MASLD or advanced liver fibrosis. Mortality data were obtained through probabilistic linkage with the National Death Index, provided by the National Center for Health Statistics (NCHS). Follow-up was conducted from the date of each participant’s baseline interview until December 31, 2019. Causes of death were classified according to the International Classification of Diseases, 10th Revision (ICD-10).

### 2.4. Assessment of covariates and confounding factors

A comprehensive set of covariates from NHANES was included in the analysis, encompassing demographic and socioeconomic variables (e.g., age, sex, race/ethnicity, education level, and poverty-to-income ratio), lifestyle factors (e.g., smoking status and physical activity), and clinical comorbidities (including diabetes, hypertension, cardiovascular disease, cancer, and asthma). Additional variables derived from physical examinations and laboratory data – such as daily energy intake (averaged from 2 24-hour dietary recalls), total fat and sugar intake, total cholesterol, and serum uric acid – were also incorporated to control for potential confounding.

### 2.5. Missing data handling

For covariates with a substantial proportion of missing values, continuous variables were converted into categorical variables according to their median values. Participants with missing observations were retained by assigning them to an additional category (“unclear”) in the regression models. This approach allowed the inclusion of individuals with incomplete covariate data and helped maintain statistical power in the analyses.

### 2.6. Statistical analysis

All statistical analyses were conducted using EmpowerStats software with R version 4.2.0. A 2-sided *P*-value < .05 was considered statistically significant. Sampling weights were recalculated for the combined NHANES cycles based on official methodological guidance.^[30]^ Weighted linear regression was applied for continuous variables, while weighted chi-square tests were used for categorical variables. Continuous variables are reported as weighted means with 95% confidence intervals (CIs), and categorical variables as weighted proportions with 95% CIs. Multivariate Cox proportional hazards models were constructed to examine the associations between CTI and both all-cause and cardiovascular mortality in the MASLD and advanced liver fibrosis cohorts. Three models were specified: model 1 was unadjusted; model 2 was adjusted for age, race, education, and poverty-to-incomeratio; and model 3 adjusted for all relevant covariates listed in Table 1. In addition, CTI was categorized into tertiles, and Kaplan–Meier survival curves were plotted to compare survival across CTI levels. To explore potential nonlinear associations, smoothed curve fitting was performed using restricted cubic spline (RCS) and penalized spline methods. If nonlinearity was detected, an inflection point was determined using the maximum likelihood approach. A 2-piecewise Cox regression model was then fitted to estimate hazard ratios on either side of the threshold. Subgroup analyses were also conducted to assess whether the association between CTI and mortality varied by key clinical subgroups.

**Table 1:** Baseline data grouped according to patient survival status.

Characteristic	Survival group	Death group	*P*-value
	N = 6985	N = 1806	
Age (yr)	49.38 (48.81, 49.94)	67.00 (66.15, 67.85)	<.0001
Serum cholesterol (mg/L)	204.20 (202.70, 205.70)	199.55 (196.64, 202.46)	.0057
BMI	33.86 (33.65, 34.07)	32.82 (32.41, 33.22)	<.0001
Serum uric acid (mg/dL)	5.87 (5.83, 5.91)	6.29 (6.21, 6.37)	<.0001
CTI	8.65 (8.56, 8.75)	8.79 (8.71, 8.87)	.0211
Gender (%)			.0121
Male	54.54 (53.13, 55.94)	58.91 (55.73, 62.02)	
Female	45.46 (44.06, 46.87)	41.09 (37.98, 44.27)	
Race (%)			<.0001
Mexican American	13.80 (11.63, 16.30)	6.18 (4.49, 8.45)	
White	69.38 (65.97, 72.60)	81.15 (77.97, 83.96)	
Black	11.42 (9.71, 13.38)	9.76 (7.92, 11.98)	
Other race	5.40 (4.58, 6.35)	2.91 (1.99, 4.23)	
Education Level (%)			<.0001
Less than high school	17.14 (15.58, 18.83)	29.78 (26.79, 32.96)	
High school	24.88 (23.20, 26.64)	28.51 (26.16, 30.99)	
More than high school	57.98 (55.93, 60.00)	41.71 (38.33, 45.16)	
Smoked (%)			<.0001
Yes	42.68 (40.93, 44.45)	62.52 (59.49, 65.44)	
No	57.32 (55.55, 59.07)	37.48 (34.56, 40.51)	
Physical activity (%)			<.0001
Never	33.04 (31.34, 34.78)	53.15 (50.01, 56.26)	
Moderate	34.44 (32.94, 35.96)	34.23 (31.26, 37.34)	
Vigorous	32.52 (30.87, 34.22)	12.62 (10.61, 14.94)	
Asthma (%)			.4569
Yes	15.28 (14.12, 16.51)	14.33 (12.25, 16.69)	
No	84.72 (83.49, 85.88)	85.67 (83.31, 87.75)	
Coronary artery disease (%)			<.0001
Yes	3.84 (3.27, 4.50)	15.62 (13.66, 17.80)	
No	96.16 (95.50, 96.73)	84.38 (82.20, 86.34)	
Cancers (%)			<.0001
Yes	9.27 (8.51, 10.09)	24.07 (21.79, 26.51)	
No	90.73 (89.91, 91.49)	75.93 (73.49, 78.21)	
Diabetes (%)			<.0001
Yes	13.44 (12.34, 14.63)	30.42 (27.97, 32.99)	
No	84.13 (82.86, 85.33)	66.11 (63.51, 68.61)	
Borderline	2.43 (1.98, 2.98)	3.47 (2.60, 4.62)	
High blood pressure (%)			<.0001
Yes	42.31 (40.71, 43.93)	66.73 (63.82, 69.51)	
No	57.69 (56.07, 59.29)	33.27 (30.49, 36.18)	
PIR (%)			<.0001
<1.3	17.97 (16.57, 19.46)	23.61 (21.28, 26.12)	
≥1.3 < 3.5	33.44 (31.63, 35.29)	42.12 (39.43, 44.85)	
≥3.5	42.37 (40.09, 44.69)	27.98 (24.91, 31.26)	
Unclear	6.23 (5.39, 7.18)	6.29 (5.06, 7.80)	
Total Kcal (%)			<.0001
Lower	37.05 (35.56, 38.58)	51.27 (47.94, 54.59)	
Higher	52.35 (50.71, 53.98)	40.22 (37.03, 43.48)	
Unclear	10.60 (9.66, 11.62)	8.51 (6.88, 10.50)	
Total sugar (%)			.0003
Lower	37.41 (35.96, 38.88)	44.17 (40.95, 47.45)	
Higher	42.24 (40.63, 43.87)	38.09 (34.44, 41.88)	
Unclear	20.35 (19.07, 21.69)	17.74 (15.18, 20.62)	
Total fat (%)			<.0001
Lower	36.55 (34.67, 38.47)	47.96 (44.52, 51.42)	
Higher	52.85 (50.84, 54.85)	43.53 (40.25, 46.86)	
Unclear	10.60 (9.66, 11.62)	8.51 (6.88, 10.50)	
Advanced liver fibrosis (%)			<.0001
No	92.85 (91.63, 93.91)	77.43 (74.78, 79.87)	
Yes	6.30 (5.43, 7.29)	21.92 (19.72, 24.29)	

BMI = body mass index, CTI = C-reactive protein–triglyceride–glucose index, PIR = poverty-to-income ratio.

## 3. Results

### 3.1. Participant characteristics

A total of 8791 individuals with MASLD from the NHANES database were included in the final analysis, comprising 4552 men and 4239 women. During the follow-up period, 1806 deaths were recorded. Baseline demographic, clinical, and biochemical characteristics stratified by survival status are summarized in Table 1. Compared to survivors, individuals in the deceased group were more likely to be older, male, White, less educated, and had higher levels of uric acid and CTI, as well as higher prevalence of hypertension, diabetes, cancer, and advanced liver fibrosis.

### 3.2. Role of CTI in predicting long-term outcomes in patients with MASLD and advanced liver fibrosis

**Figure 2.**
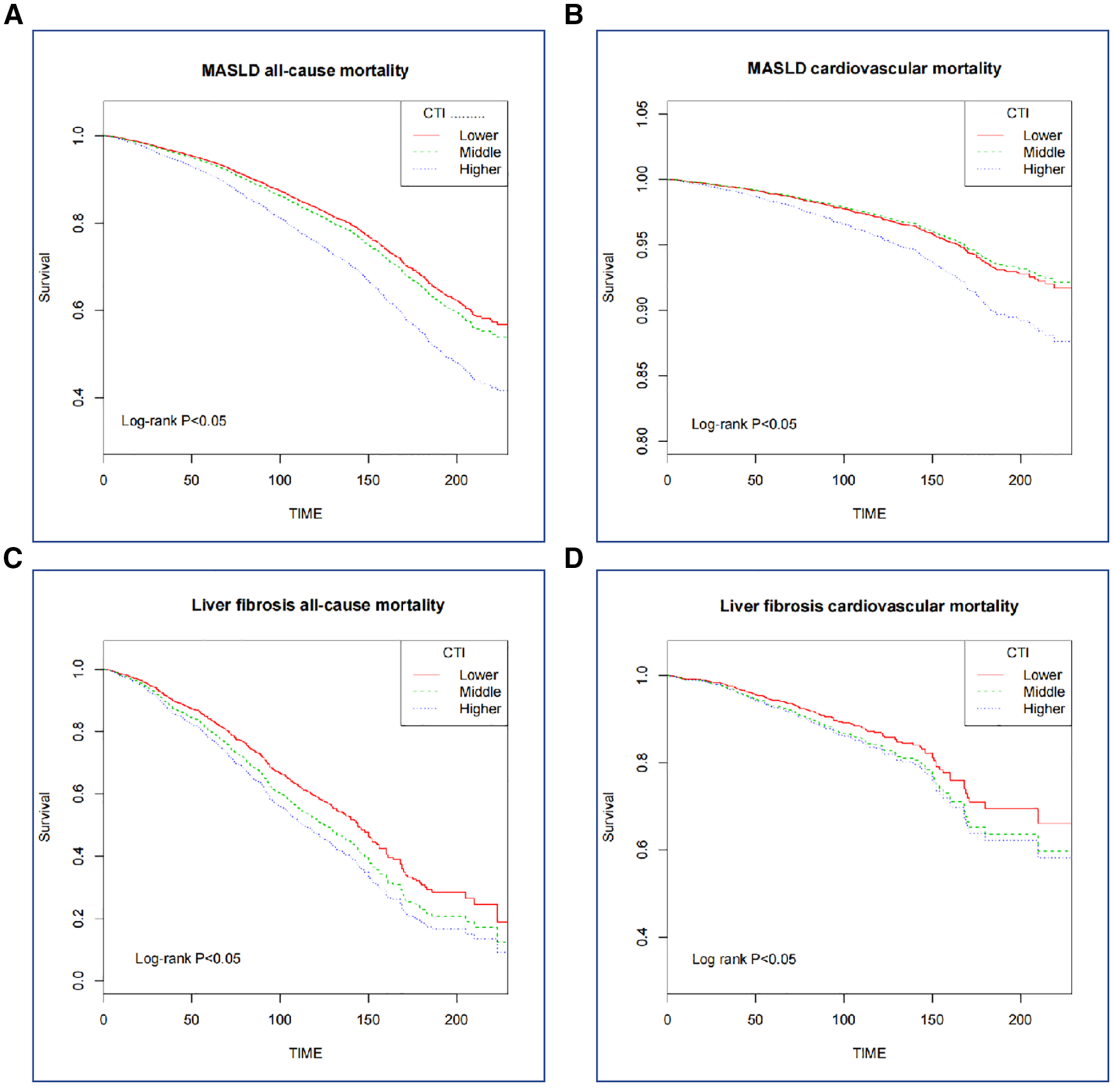
Kaplan–Meier survival curves for all-cause and cardiovascular mortality among patients with MASLD and advanced liver fibrosis, stratified by CTI tertiles. (A) All-cause mortality in the MASLD population; (B) cardiovascular mortality in the MASLD population; (C) all-cause mortality in patients with advanced liver fibrosis; (D) cardiovascular mortality in patients with advanced liver fibrosis. Participants were categorized into CTI tertiles: low (red solid line), middle (green dashed line), and high (blue dotted line). Higher CTI levels were associated with lower survival probabilities. Log-rank tests showed significant differences among groups in all panels (*P* < .05). CTI = C-reactive protein –Triglyceride–Glucose Index, MASLD = metabolic dysfunction-associated steatotic liver disease.

Kaplan–Meier survival curves were used to examine the association between CTI and both all-cause and cardiovascular mortality in MASLD and advanced liver fibrosis populations. As shown in Figure 2, individuals with higher CTI levels exhibited significantly lower survival probabilities. These associations remained significant in both MASLD and advanced liver fibrosis subgroups (log-rank test, all *P* < .05).

Cox proportional hazards regression was employed to assess the prognostic impact of CTI in MASLD and advanced liver fibrosis populations. In the fully adjusted model (model 3), higher CTI was significantly associated with an increased risk of all-cause (HR = 1.16, 95% CI: 1.12–1.20) and cardiovascular mortality (HR = 1.12, 95% CI: 1.04–1.20) among MASLD patients. Similarly, in the advanced fibrosis subgroup, elevated CTI remained significantly associated with all-cause mortality (HR = 1.14, 95% CI: 1.07–1.23) and cardiovascular mortality (HR = 1.14, 95% CI: 1.01–1.29). When CTI was categorized into tertiles, individuals in the highest tertile exhibited a significantly higher risk of all-cause mortality; a similar but statistically nonsignificant upward trend was observed for cardiovascular mortality (Table 2).

**Table 2:** COX regression analysis of CTI with all-cause and cardiovascular disease mortality in patients with MASLD and advanced liver fibrosis.

Characteristic	Model 1 HR (95%CI)	Model 2 HR (95%CI)	Model 3 HR (95%CI)
All-cause mortality in the MASLD group			
CTI	1.09 (1.06, 1.13)	1.22 (1.18, 1.27)	1.16 (1.12, 1.20)
Categories			
Lower	1	1	1
Middle	0.95 (0.85, 1.06)	1.21 (1.08, 1.35)	1.13 (1.01, 1.26)
Higher	1.33 (1.18, 1.49)	1.88 (1.68, 2.12)	1.61 (1.43, 1.82)
Cardiovascular mortality in the MASLD group			
CTI	1.09 (1.02, 1.17)	1.13 (1.05, 1.21)	1.12 (1.04, 1.20)
Categories			
Lower	1	1	1
Middle	1.05 (0.84, 1.31)	1.15 (0.92, 1.43)	1.17 (0.92, 1.49)
Higher	1.14 (0.93, 1.41)	1.29 (1.03, 1.61)	1.23 (0.96, 1.58)
All-cause mortality in the liver fibrosis group			
CTI	1.00 (0.94, 1.06)	1.15 (1.07, 1.22) < 0.0001	1.14 (1.07, 1.23)
Categories			
Lower	1	1	1
Middle	0.98 (0.79, 1.22)	1.27 (1.02, 1.58)	1.25 (1.00, 1.57)
Higher	0.85 (0.68, 1.07)	1.47 (1.16, 1.88)	1.45 (1.12, 1.86)
Cardiovascular mortality in the liver fibrosis group			
CTI	0.97 (0.87, 1.08)	1.15 (1.03, 1.29) 0.0146	1.14 (1.01, 1.29)
Categories			
Lower	1	1	1
Middle	0.84 (0.56, 1.25)	1.24 (0.82, 1.85)	1.24 (0.81, 1.89)
Higher	0.75 (0.50, 1.13) 7	1.50 (0.98, 2.30)	1.35 (0.85, 2.13)

Model 1 was adjusted for no covariates.

Model 2 was adjusted for age, race, and education.

Model 3 was adjusted for all covariates.

CI = confidence interval, CTI = C-reactive protein–triglyceride–glucose index, HR= hazard ratio, MASLD = metabolic dysfunction-associated steatotic liver disease.

### 3.3. Restricted cubic spline and threshold analysis

**Figure 3.**
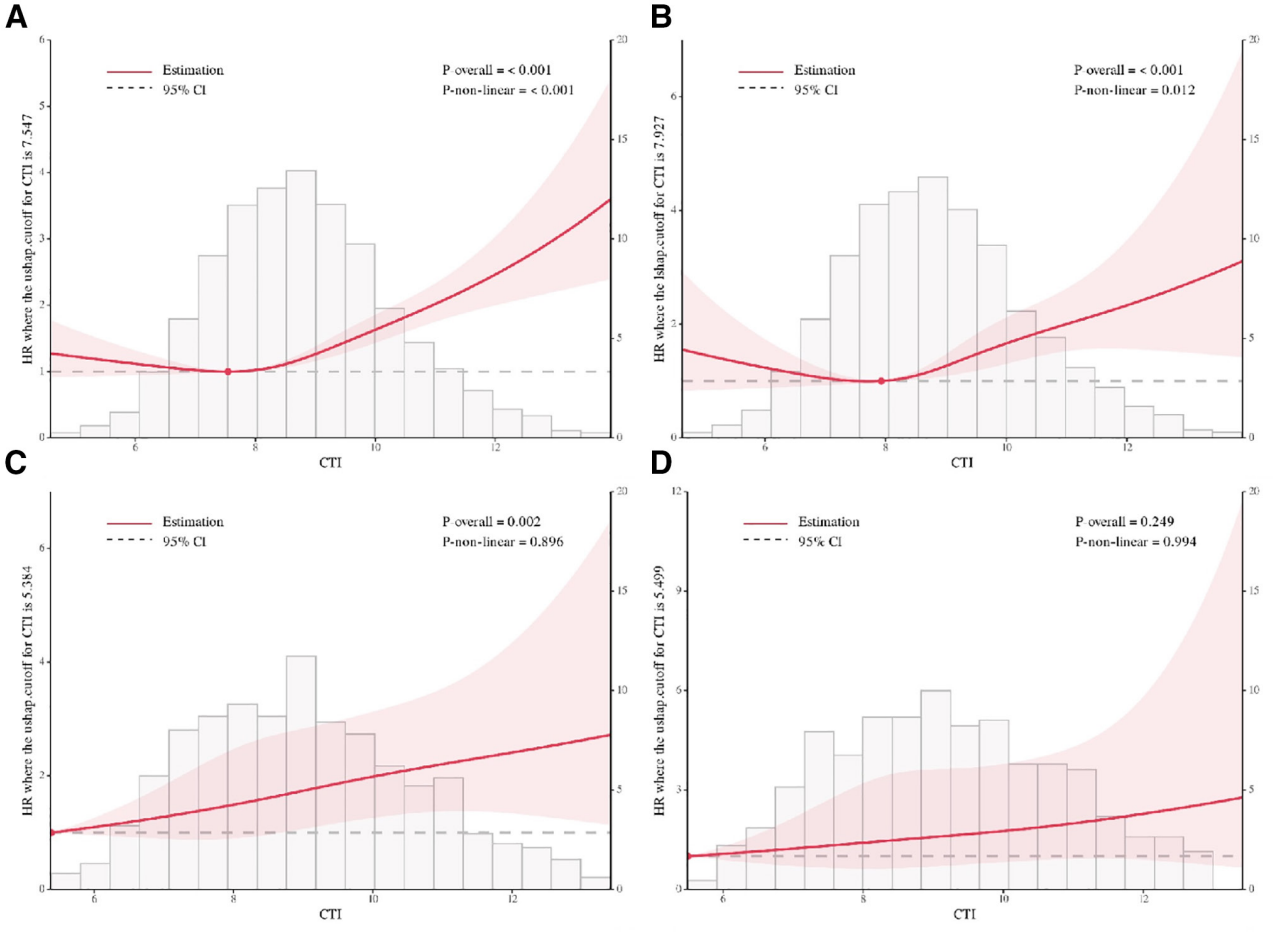
Restricted cubic spline (RCS) curves for the association between CTI and all-cause or cardiovascular mortality in MASLD and advanced liver fibrosis populations. (A) All-cause mortality in the MASLD population; (B) cardiovascular mortality in the MASLD population; (C) all-cause mortality in the advanced liver fibrosis population; (D) cardiovascular mortality in the advanced liver fibrosis population. Hazard ratios (HRs) and 95% confidence intervals (shaded area) were estimated using weighted Cox proportional hazards models. The red solid line represents the fitted nonlinear association, while the gray dashed line indicates the reference level (HR = 1). Histogram bars represent the distribution of CTI. CTI = C-reactive protein –Triglyceride–Glucose Index, HR = hazard ratio, MASLD = metabolic dysfunction-associated steatotic liver disease, RCS = restricted cubic spline.

Among MASLD patients, RCS modeling revealed a nonlinear, “U”-shaped association between CTI and all-cause mortality (*P* for nonlinearity < .001). Mortality risk declined until a CTI of approximately 7.547, after which it rose again with increasing CTI values. A similar “U”-shaped trend was observed for cardiovascular mortality (*P* for nonlinearity = .012), with the lowest risk occurring at a CTI of 7.927. Both very low and very high CTI levels were associated with elevated mortality risks (Fig. 3).

In contrast, among patients with advanced liver fibrosis, a linear relationship was observed between CTI and all-cause mortality (*P* for nonlinearity = .896), with the lowest risk at a CTI of approximately 5.384 and a steady increase thereafter. For cardiovascular mortality, a linear trend was also noted (*P* for nonlinearity = .994), with the lowest risk at a CTI of 5.499 and a gradual rise thereafter (Fig. 3).

### 3.4. Subgroup analyses

**Figure 4.**
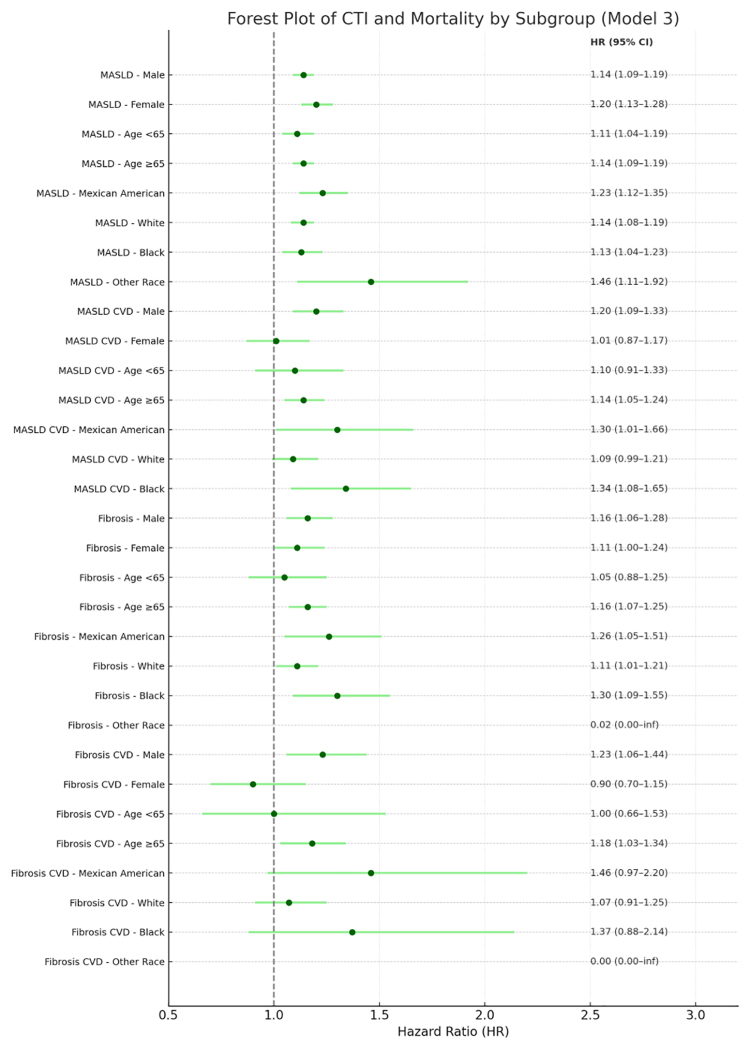
Forest plot of the association between CTI and all-cause or cardiovascular mortality across subgroups in MASLD and advanced liver fibrosis populations (model 3). CTI = C-reactive protein –Triglyceride–Glucose Index, CVD = cardiovascular disease, MASLD = metabolic dysfunction-associated steatotic liver disease.

Subgroup analyses were performed to explore heterogeneity in the association between CTI and all-cause mortality in MASLD and advanced liver fibrosis populations (Fig. 4). Among MASLD patients, the association remained significant across multiple subgroups in the fully adjusted model. Notably, elevated CTI was significantly associated with all-cause mortality in females (HR = 1.20, 95% CI: 1.13–1.28), individuals aged ≥65 years (HR = 1.14, 95% CI: 1.09–1.19), Mexican Americans (HR = 1.23, 95% CI: 1.12–1.35), Whites (HR = 1.14, 95% CI: 1.08–1.19), African Americans (HR = 1.13, 95% CI: 1.04–1.23), and other racial groups (HR = 1.46, 95% CI: 1.11–1.92).

Among patients with advanced liver fibrosis, elevated CTI was also significantly associated with higher all-cause mortality across various subgroups (Fig. 4). This association was significant in both men (HR = 1.16, 95% CI: 1.06–1.28) and women (HR = 1.11, 95% CI: 1.00–1.24), as well as in participants aged≥65 years (HR = 1.16, 95% CI: 1.07–1.25). Stratified analyses by race/ethnicity revealed statistically significant associations among Mexican Americans (HR = 1.26, 95% CI: 1.05–1.51), Whites (HR = 1.11, 95% CI: 1.01–1.21), and African Americans (HR = 1.30, 95% CI: 1.09–1.55). These findings suggest a consistent prognostic relevance of CTI across diverse patient subgroups.

## 4. Discussion

This study is the first to systematically evaluate the prognostic value of the CTI for long-term outcomes in patients with MASLD and advanced liver fibrosis, using a nationally representative cohort from NHANES 2001 to 2018. Our findings demonstrate that CTI is significantly associated with both all-cause and cardiovascular mortality. In MASLD patients, the relationship followed a characteristic “U”-shaped curve, whereas in those with advanced liver fibrosis, the association was linear and increasing. Notably, the prognostic relevance of CTI was particularly prominent in women, individuals aged ≥ 65 years, and racial/ethnic minorities, underscoring its potential utility in identifying high-risk subgroups.

Among MASLD patients, the observed “U”-shaped association between CTI and mortality risk may reflect a complex interplay between metabolic and inflammatory states. In contrast, high CTI levels reflect persistent inflammation and insulin resistance, both of which accelerate hepatic injury and cardiovascular complications. Previous studies have also demonstrated that the TyG index, a component of CTI, is significantly associated with hepatic steatosis and liver fibrosis. A cross-sectional study in China reported a strong positive correlation between TyG and fibrosis severity in NAFLD patients, with those in the highest quartile showing significantly elevated fibrosis risk (OR = 1.98, 95% CI: 1.33–2.22).^[31]^ Likewise, an NHANES-based US study confirmed that TyG was linearly associated with ≥F2 fibrosis in MAFLD patients.^[32]^ Similarly, CRP – a prototypical inflammatory biomarker – is consistently associated with disease severity in MASLD and liver fibrosis. Wu et al demonstrated that elevated hs-CRP levels were linked to significant or progressive fibrosis in a large cohort from China and the United States, with a nonlinear dose–response pattern.^[33]^ Korean studies have also shown increased hs-CRP in patients with ≥F2 fibrosis,^[34]^ and in obese MASLD populations, hs-CRP exhibited predictive capacity for fibrosis (OR = 2.63, 95% CI: 1.07–6.48).^[35]^ Furthermore, chronic inflammation and insulin resistance are interdependent drivers of MASLD progression. CRP has been shown to induce insulin resistance via disruption of insulin signaling and to promote hepatic inflammation by activating Kupffer cells and macrophages.^[36,37]^ M1-type macrophages further contribute to hepatic fibrosis by stimulating hepatic stellate cells through cytokines such as TNF-α, IL-6, and IL-1β, aggravating immunometabolic imbalance.^[10,11]^

In patients with advanced liver fibrosis, a linear relationship was observed between CTI and mortality risk. Unlike early-stage MASLD, individuals with advanced fibrosis may experience loss of metabolic compensation and immune adaptation. In this setting, persistent insulin resistance and chronic inflammation jointly promote the development of extrahepatic complications, including adverse cardiovascular outcomes and elevated all-cause mortality.^[38,39]^ The linear increase in cardiovascular mortality with rising CTI suggests that this index may act as a continuous pathogenic driver in this population. Supporting this, studies in patients with nonalcoholic steatohepatitis (NASH) have identified cardiovascular events as a leading cause of mortality in advanced liver disease.^[38]^ Additionally, insulin resistance plays a central role in linking hepatic fibrosis to systemic metabolic dysregulation and multi-organ damage.^[39]^ In conclusion, CTI, as a novel composite indicator integrating inflammation and metabolic status, showed significant associations with mortality risk at different stages of MASLD progression, and the mechanism may be related to the combined effects of inflammatory factor-driven, insulin signaling inhibition and imbalance of immune-metabolic network. This index has broad clinical application in risk prediction and precise stratification.

The present study possesses several notable strengths. First, it utilizes data from the nationally representative NHANES 2001 to 2018 cohort, which offers a large sample size with strong external validity and scientific rigor. Second, this is the first study to apply the CTI – a composite marker of C-reactive protein and the TyG index – to evaluate long-term mortality risk in individuals with MASLD and advanced liver fibrosis. This underscores CTI’s potential as a clinically useful tool for comprehensively assessing systemic inflammation and metabolic dysfunction. Furthermore, the relationships between CTI and both all-cause and cardiovascular mortality were systematically analyzed using multivariate Cox regression, subgroup analyses, and RCS modeling to explore both linear and nonlinear associations. The results were robust and statistically rigorous. However, several limitations should be acknowledged. As a retrospective observational study, causal inference is limited, and residual confounding cannot be entirely excluded. CTI was derived from a single-time-point blood sample, which may not accurately represent long-term inflammatory or insulin resistance status. The diagnoses of MASLD and hepatic fibrosis were based on noninvasive serologic algorithms (e.g., FLI, NFS, FIB-4), lacking confirmatory histological or imaging evidence, which may introduce misclassification bias. Additionally, cause-of-death information was based on ICD codes, which could underestimate the multifactorial nature of mortality. Furthermore, handling missing covariates by creating an “unclear” category and the absence of formal proportional hazards assumption testing for the Cox models may introduce residual bias and methodological limitations. Additionally, the clinical relevance of the U-shaped association in MASLD should not be overstated; extremely low CTI values may reflect unmeasured heterogeneous conditions, such as underlying malnutrition or severe chronic illnesses, rather than a protective metabolic state. Despite these limitations, this study provides a valuable foundation for the potential clinical application of CTI in metabolic liver disease risk assessment, warranting further validation in prospective cohort studies.

## 5. Conclusion

This study, based on a large US NHANES cohort, found that elevated CTI – a marker of systemic inflammation and insulin resistance – was significantly associated with increased all-cause and cardiovascular mortality in individuals with MASLD and advanced liver fibrosis. A “U”-shaped relationship was observed in MASLD patients, and a linear trend in those with advanced fibrosis. These associations were stronger in women, older adults, and ethnic minorities. CTI may serve as a simple tool for risk stratification in MASLD and warrants validation in future prospective studies.

## Author contributions

**Conceptualization:** Hao Wang.

**Data curation:** Hao Wang.

**Formal analysis:** Yan Wang.

**Investigation:** Hao Wang.

**Methodology:** Yifeng Zhou, Yuan Zhou.

**Project administration:** Yuan Zhou.

**Software:** Yifeng Zhou.

**Visualization:** Xiaoyu Cui.

**Writing – original draft:** Hao Wang, Yifeng Zhou, Xiaoyu Cui, Yan Wang.

**Writing – review & editing:** Yuan Zhou.
